# Single-cell RNA-seq reveals the communications between extracellular matrix-related components and Schwann cells contributing to the earlobe keloid formation

**DOI:** 10.3389/fmed.2022.1000324

**Published:** 2022-10-26

**Authors:** Taogen Gong, Yayu Wang, Shaowei Dong, Xiaoshi Ma, Danfeng Du, Chang Zou, Qijun Zheng, Zhong Wen

**Affiliations:** ^1^Otolaryngology-Head and Neck Surgery Center, Zhujiang Hospital, Southern Medical University, Guangzhou, China; ^2^Department of Otolaryngology-Head and Neck Surgery, The Second Clinical Medical College, Jinan University (Shenzhen People’s Hospital), Shenzhen, China; ^3^Department of Cardiovascular Surgery, The Second Clinical Medical College, Jinan University (Shenzhen People’s Hospital), Shenzhen, China; ^4^School of Medicine, Life and Health Sciences, The Chinese University of Hong Kong, Shenzhen, China; ^5^Department of Pathology, The Second Clinical Medical College, Jinan University (Shenzhen People’s Hospital), Shenzhen, China

**Keywords:** scRNA-seq, earlobe keloid, Schwann cells, ECM-related populations, cell interactions, site-specific pathogenesis

## Abstract

Keloid is a major type of skin fibrotic disease, with one prominent feature of extensive accumulation of extracellular matrix (ECM) components, and another feature of pain/itching, which is closely related to the peripheral nervous system (PNS). However, the molecular pathogenesis of these two prominent features still needs to be further explored. In the present study, we performed single-cell RNA sequencing (scRNA-seq) on clinical earlobe keloid samples and adjacent normal skin samples and constructed a keloid atlas of 31,379 cells. All cells were clustered into 13 major cell types using cell-type-specific markers. Among them, fibroblast, vascular endothelial cells, and smooth muscle cells were defined as the ECM-related populations according to their ECM-associated functions. Also, we found that Schwann cells (SCs) were the main neuron cells of PNS in the skin. Interestingly, the cell proportions of ECM-related populations, as well as SC were increased significantly in the earlobe keloid compared to the adjacent normal tissues, suggesting an important role of these cell types in the development of the earlobe keloid. Comprehensive cell–cell interaction analysis at the single-cell level revealed a strong interaction between SC and ECM-related subgroups which might be mediated by SEMA3C signaling pathways and MK/PTN gene family, which are found to be mainly involved in promoting cell proliferation and migration. Moreover, further exploration of the interactions of ECM-related populations and SC in different keloids, including earlobe keloid, back keloid, and chest keloid revealed an increasing amount of TGFβ–TGFβ receptor interactions in chest/back keloids as compared to earlobe keloid, which suggested the anatomic site-specific pathogenesis in different keloids. Altogether, these findings suggested the interactions between ECM-related populations and SC contributing to the earlobe keloid formation and helped us to better understand the pathogenesis of keloids.

## Introduction

Keloids are benign fibroproliferative tumors in the dermis, but they are unable to metastasize. After skin wounding, the self-healing mechanism of the skin will be triggered and produced scars during the wound healing process. However, when this wound healing process has been disturbed, it can result in excessive scar formation ranging from hypertrophic scars to keloids (1). As keloid pathogenesis is still unclear, keloid treatments are commonly through surgical resection, radiotherapy, and local injection of glucocorticoid, etc. However, these treatments still have a high recurrence rate and tend to aggravate the disease after recurrence, making it more difficult to treat keloid (2–4). Therefore, a thorough understanding of keloid pathogenesis would facilitate the development of clinical treatments for this disease.

Keloids tend to occur in areas of high mobility and tension, such as the chest, scapular region, earlobes, and upper arm (5). Keloids from different anatomical sites have site-specific morphology. For example, the single back keloids are grape-shaped, chest keloids are butterfly or non-butterfly shaped, and earlobe keloids have reniform to bulbous shapes (5). Due to different body surface areas suffering kinds of influences, such as UV radiation, trauma, and mechanical forces (6, 7), the occurrence and pathogenesis of keloids are also site-specific, including the difference between extracellular matrix (ECM) components and immune system (7).

The most prominent features of keloids are fibroblast proliferation and an extensive accumulation of ECM components, such as collagen (8). Now, a lot of studies mainly focus on the role of fibroblast in keloid pathogenesis (8–10). However, another prominent clinical feature of keloids is pain and itching, which leads to patients feeling very painful and increases their psychological pressure. This feature is closely related to the abundance of the peripheral nervous system (PNS) in the skin tissue. Skin as the largest organ of the human body contains an intricate PNS. The main cellular component of PNS is remarkable plastic glia, Schwann cells (SCs). In healthy skin, SC performs functions through differentiated (myelinating) or undifferentiated (non-myelinating) states (11). However, the wound microenvironment could reprogram SC to invasive mesenchymal-like cells to drive axonal regrowth, cell proliferation, and inflammatory response to promote wound healing (12). Furthermore, in mice skin wound models, SC has been shown to dedifferentiate and regulate myofibroblast differentiation via TGF-β to promote skin wound healing (13). However, there is a lack of direct studies on the role of SC in earlobe keloid formation, and most studies are mainly based on mice models, lacking direct clinical samples.

In our study, we used scRNA-seq to characterize the transcriptomic profiles of single cells in earlobe keloid samples and adjacent normal skin samples. We further studied the interactions between ECM-related populations and SC in the development of keloids. In addition, we found that different kinds of keloids contribute to ECM accumulation by different mechanisms. These results suggested that the specific interactions between ECM-related subpopulations and Schwann cells could contribute to earlobe keloid formation.

## Materials and methods

### Patient specimens and tissue dissociation

This study was approved by the Medical and Ethics Committees of Shenzhen People’s Hospital and each patient signed informed consent before enrolling in this study. We had a total of five patients, and we collected the keloid tissue and corresponding normal skin tissue of each patient. Normal skin tissue is adjacent to relatively normal tissue to keloid tissue. However, we only collected single-cell transcriptome data from a control sample for a technical reason. Detailed information is shown in Supplementary Table 1. No patient received chemotherapy, radiotherapy, or intralesional steroid treatment before surgery. The skin tissues were washed two times in PBS and cut into small pieces to further disassociated in the lyase cocktail at 37°C for 30 min. Single-cell suspension was obtained by filtration through a 40-μm filter. Red blood cells were removed with lysis buffer (Thermo Fisher Scientific, cat. no. 1966634). Cell viability were detected by trypan blue staining (Thermo Fisher Scientific, Waltham, MA, USA, cat. no. T10282). If the cell viability is less than 80%, live cells would be further purified by a Dead Cell Removal Kit (Miltenyi Biotec, GER, cat. no. 130-090-101).

### Single-cell library preparation and sequencing

Single-cell gel beads-in-Emulsion (GEM) generation, barcoding, sample cleanup, cDNA amplification, and cDNA library construction were performed using Chromium Next GEM Single Cell 3’ Reagent Kits v3.1 (PN-1000121). After single-cell library preparation, the final library was quality-tested by Agilent 2100 bioanalyzer and sequenced on a Hiseq-PE150 platform (Novogene Bioinformatics Technology Co. Ltd, Beijing, China).

### Single-cell RNA sequencing data preprocessing

The raw FASTQ sequence reads were preprocessed by Cell Ranger software (Version 5.0.1, V5.0.1). Reads were aligned to prebuilt GRCh38 reference assembly (V3.0.0, 10 × Genomics) and unique molecular identifier (UMI) counting was identified by cellranger count for each sample, and then UMI counting matrices were outputted and processed by R package Seurat (V4.0.5) (14) for downstream analysis. In order to filter low-quality cells and cell doublets, we performed a stringent quality control for each sample based on violin plots (Supplementary Figure 1), such as excluded cells with >15% mitochondrial UMIs and >1% red blood cells, detailed filtering parameters are shown in Supplementary Table 2. And then we used the Seurat function NormalizeData (parameters: normalization.method = “LogNormalize,” scale.factor = 10,000) to normalize UMI counts for each cell by total expression, which can reduce batch effect between samples. We then used Seurat function FindVariableFeatures to define the top 2,000 highly variable genes (HVGs) and integrated all samples with a standard Seurat V5 integration algorithm based on 2,000 HVGs for batch effect correction.

### Dimensional reduction and clustering

We ran a principal component analysis (PCA) dimensionality reduction using Seurat function RunPCA and used Seurat function ElbowPlot to identify the significant dimensions. In our study, we selected 30 PCs for clustering. Before clustering the cells, we constructed a shared nearest neighbor (SNN) graph based on the Euclidean distance in PCA space using Seurat function FindNeighbors. Cells were then clustered using Seurat function FindClusters based on the Louvain algorithm, with a resolution of 1.5. Cells were displayed on the Unsupervised Uniform Manifold Approximation and Projection (UMAP) plot. Marker genes for each cluster were selected using Seurat function FindAllMarkers (logfc.threshold = 0.25, test.use = “wilcox”) and included canonical marker genes that had been reported before.

### Phylogenetic analysis

We used Seurat function BuildClusterTree to construct a phylogenetic tree relating the “average” cell from each cell lineage. In addition to setting dim = 1:30, we used the default parameters.

### Subpopulation analysis

After defining the major cell types, we selected some for further annotation, including fibroblast (FB), endothelial cells (ECs), and smooth muscle cells (SMCs). As we clustered all cells at a high resolution (resolution = 1.5), we directly split a UMI count matrix of cells in each major cell population from the whole matrix and performed the analysis to identify cell subpopulations. The steps of subpopulation analysis were similar to those of the whole dataset.

### Functional analysis

Gene ontology (GO) functional enrichment (15) of differentially expressed genes (DEGs) was performed on R package clusterProfiler V4.2.0 (16) and a cutoff *P*-value < 0.05 was used to filter the significant enrichment results.

### Trajectory and cell cycle analysis

We used the R package monocle (V2.21.1) (17) to infer the pseudotime trajectories of cells in specific subpopulations. The UMI matrix, gene information, and phenotype information were extracted from Seurat objects directly for building traces. Branches in the cell trajectory represent cells in different states with alternative gene expression patterns.

### Cell–cell interaction network analysis

We used the R package CellChat (V1.1.3) (18) to identify the potential interactions between different cell populations. The CellChatDB human database was imported to construct the ligand–receptor interactions and secreted signaling was used for cell–cell communication analysis.

### Statistical analysis

All statistical analyses were performed in R (V4.1.2). Student’s *t*-test or Wilcoxon rank-sum test were used for the comparison of the two groups. P were adjusted for multiple hypothesis testing using the Benjamini–Hochberg method. A *P*-value < 0.05 was considered statistically significant.

## Results

### Single-cell transcriptomic map and cellular alterations in earlobe keloid

**FIGURE 1:**
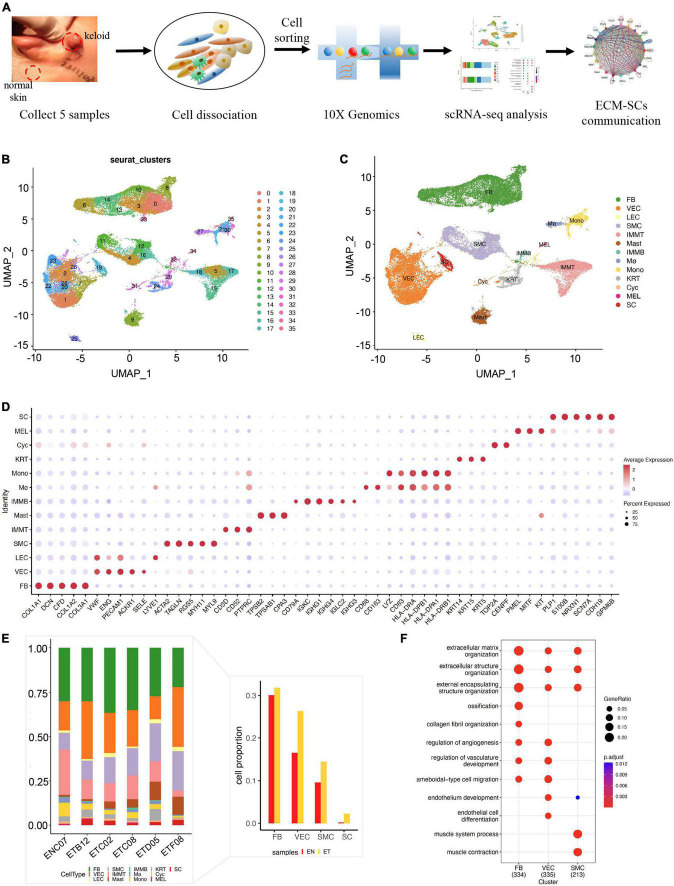
Single-cell RNA-seq revealed the cell landscape of normal skin and earlobe keloid samples. **(A)** The workflow of scRNA-seq in human normal skin (EN) and earlobe keloid samples (ET). **(B)** All cells were merged and clustered by UMAP-clustering and were divided into 35 clusters. **(C)** All cells were annotated into 13 major cell types, including: fibroblast (FB), endothelial cell (EC), smooth muscle cell (SMC), T cell (IMMT), B cell (IMMB), monocyte (Mono), macrophage (Mø), mast cell (Mast), keratinocyte (KRT), cycling cell (Cyc), melanocytes (MELs), and Schwann cells (SCs). **(D)** Dot plot shows the classical markers of 13 major cell types. Dot size presents the proportion of cells within the group expressing each gene, and dot color is related to its expression level. **(E)** Bar plot showed the cell proportions of different cell types in each sample. The right bar plot showed the cell proportions of ECM-related populations and SC between normal skin sample (EN) and earlobe keloid samples (ET). **(F)** Gene ontology (GO) biological process enrichment analysis of upregulated genes in each ECM-related population (Wilcoxon rank-sum test, Bonferroni correction, log2 fold change (log2FC) cutoff of 0.25, and adjusted *P*-value of < 0.05).

In this study, we first created a single-cell landscape of human normal skin and keloid samples by scRNA-seq. We collected five keloid tissues and one normal skin tissue from five female patients’ ears. Next, we isolated skin tissues by enzymatic digestion, detected by trypan blue staining for viability, and performed scRNA-seq on the 10× Genomics Chromium platform (Figure 1A). After preprocessing raw sequence reads and stringent quality control (Supplementary Figure 1), we used the Seurat integration algorithm to remove potential technical bias to further correct batch effect across different samples (Supplementary Figure 3A). After then, we obtained 31,379 cells (normal skin: 2,514, keloid: 28,865, Supplementary Table 3) and merged them. All cells were clustered by UMAP-clustering and were divided into 35 cell clusters (Figure 1B), which were partitioned into 13 major cell types (Figure 1C) by classic markers and particular transcriptional signatures (Figure 1D and Supplementary Figure 3B), including COL1A1 and DCN for fibroblast (FB), ENG, and PECAM1 for vascular endothelial cells (VECs), VWF, and LYVE1 for lymphatic endothelial cell (LEC), ACTA2, and TAGLN for smooth muscle cells (SMCs), CD3D for T cells (IMMT), TPSB2, and TPSAB1 for mast cells (Mast), CD79A for B cells (IMMB), CD83 and HLA-DRA for monocyte (Mono), CD163 and CD68 for macrophage (Mø), KRT14 and KRT15 for keratinocyte (KRT), TOP2A and CENPF for cycling cells (Cyc), PMEL for melanocytes (MELs), and NRXN1 and S100B for Schwann cells (SCs) (19–21).

Next, we sought to determine the major cellular changes upon the onset of keloid by calculating the proportion of each cell type in normal skin and earlobe keloid samples (Figure 1E). We found that the cell proportions of FB, VEC, and SMC were increased in keloid samples when compared to normal skin, which was consistent with previous reports (20, 22). Gene functions of these populations were associated with the ECM organization and extracellular structure organization (Figure 1F), so we defined them as ECM-related populations. Besides, it was worth mentioning that the cell proportion of SC was also increased in keloid samples (Figure 1E), and this cell type may be strongly associated with pain and itching of the keloid (23, 24).

Therefore, we further studied ECM-related populations and Schwann cells to reveal their roles in the formation of earlobe keloid.

### Characteristics of extracellular matrix-related populations and Schwann cells in earlobe keloid

**FIGURE 2:**
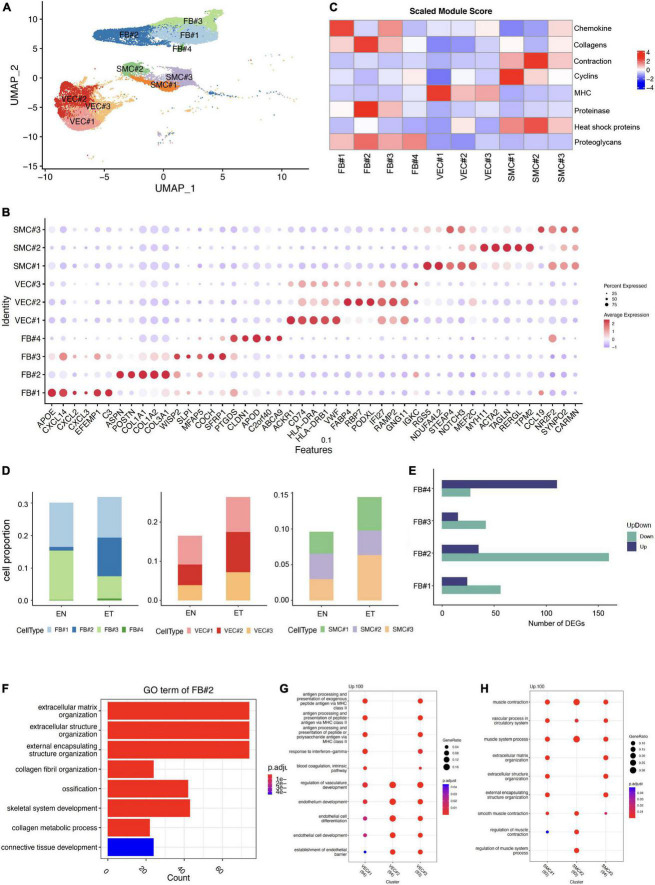
Characteristics of extracellular matrix (ECM)-related populations in earlobe keloid. **(A)** ECM-related populations were further divided into different subpopulations, including FB (FB#1, FB#2, FB#3, FB#4), VEC (VEC#1, VEC#2, VEC#3), and SMC (SMC#1, SMC#2, SMC#3). **(B)** Module scores of eight features (or functions) in each ECM-related subpopulation. **(C)** Dot plot shows the classical markers of each ECM-related subpopulation. Dot size presents the proportion of cells within the group expressing each gene, and dot color is related to its expression level. **(D)** The cell proportions of ECM-related subpopulation between normal skin sample (EN) and earlobe keloid samples (ET). **(E)** Number of DEGs in each fibroblast subpopulations between normal skin (EN) and earlobe keloid samples (ET) (Wilcoxon rank-sum test, Bonferroni correction, log2 fold change (log2FC) cutoff of 0.25, and *P*-value of < 0.05). Purple bars indicate upregulated genes, and green bars indicate downregulated genes in earlobe keloid. **(F)** Gene ontology (GO) biological process enrichment analysis of upregulated DEGs in mesenchymal fibroblast (FB#2) between earlobe keloid and normal skins. Its gene functions were mainly related to ECM organization, ossification, and skeletal system development. **(G)** GO biological process enrichment analysis of each VEC subpopulation. **(H)** GO biological process enrichment analysis of each SMC subpopulation.

As ECM-related populations showed high heterogeneity in earlobe keloid, we further divided them into different subpopulations by distinct signatures, including FB (FB#1, FB#2, FB#3, FB#4), VEC (VEC#1, VEC#2, VEC#3), and SMC (SMC#1, SMC#2, SMC#3) (Figures 2A,B). Eight features were evaluated in each ECM-related subpopulation to further understand their gene functions (Figure 2C).

According to previously identified fibroblast subpopulation markers (20, 21, 25) and specific gene expressions of each subpopulation (Figure 2B), we could also annotate our fibroblasts into four subgroups. The dot plot of gene expression showed that FB#1: pro-inflammatory had higher cytokine gene expression (CXCL14) but lower expression of collagens (COL1A1 and COL1A2), and FB#1: pro-inflammatory had the highest module score in chemokine (Figure 2C). FB#2: mesenchymal highly expressed the three known markers, such as POSTN, COL11A1, ASPN of mesenchymal fibroblasts (20, 21, 25). Module scores also showed that FB#2: mesenchymal played well in collagens and proteinase (Figure 2C). The high expression of WISP2 and MFAP5 in FB#3: secretory-reticular and the high expression of PTGDS in FB#4: secretory-papillary, respectively, were also classic markers of each subpopulation (20, 21, 25). Next, we compared the cell proportion of each fibroblast subpopulation between keloid and normal skin samples. Results showed that FB#2: mesenchymal increased largely in keloid samples (Figure 2D) and had relatively large numbers of DEGs (Figure 2E) among the four subpopulations. Besides, the GO term of FB#2: mesenchymal showed that it was mainly related to ECM organization, ossification, and skeletal system development (Figure 2F), which could promote keloid formation. These results suggested that mesenchymal fibroblasts (FB#2) played an important role in the process of keloid by changing the cell proportion and gene function. And mesenchymal fibroblasts (FB#2) had a stronger mesenchymal component than other subpopulations.

Vascular endothelial cells were divided into three subpopulations by specific markers (Figure 2B) and gene functions (Figures 2C,G). We found that VEC#1 cluster highly expressed major histocompatibility complex (MHC) genes compared with other subpopulations, such as HLA-DRA and HLA-DRB. And it got the highest score of the MHC module among all ECM-related subpopulations (Figure 2C). According to the GO enrichment analysis, VEC#1 cluster may perform immune functions at a condition, like actively involved with antigen processing and presentation and response to interferon-gamma (Figure 2G). VEC#2 cluster mainly performed the function of endothelial cell differentiation and endothelium development, and it had the largest expansion in the earlobe keloid compared with normal skin. VEC#3 cluster contained the above two characteristics. This cluster may stay in a transitional state.

We also identified three subpopulations of SMC, which were also defined by specific markers (Figure 2B) and gene functions (Figures 2C,H). SMC#1 highly expressed RGS5, which is a specific marker of myofibroblast (26), and it got a higher cyclin module score but a lower contractile score than SMC#2 cluster. SMC#2 cluster highly and specifically expressed ACTA2 and MYH11, which are involved in smooth muscle actin and myosin (27). However, the SMC#3 cluster showed both lower cyclin gene expression and contraction gene expression and expressed higher levels of cytokine genes, such as CCL19 and IL32, which suggested it was at a stressed state (28).

**FIGURE 3:**
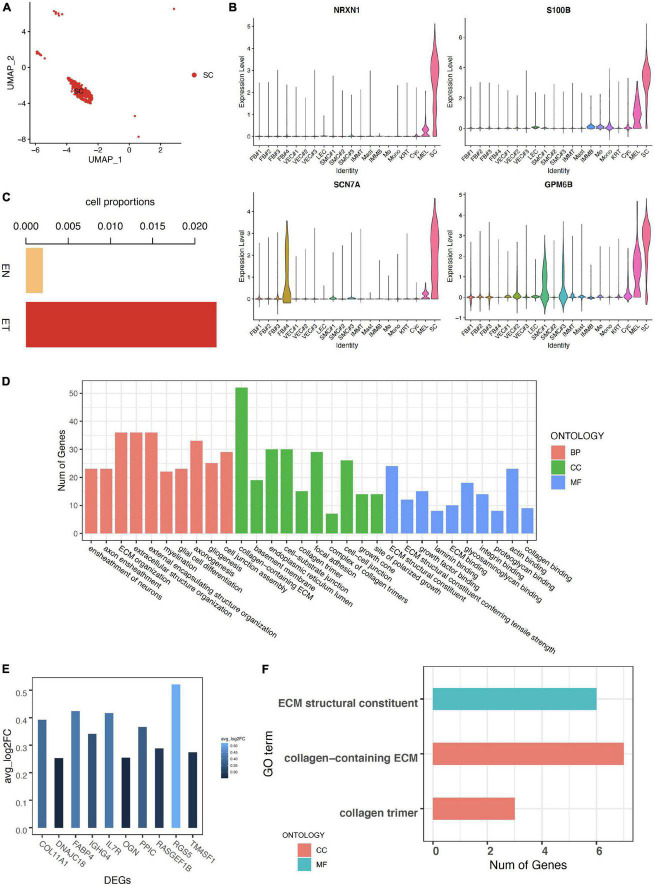
Characteristics of Schwann cells in earlobe keloid. **(A)** Uniform Manifold Approximation and Projection (UMAP) plots of Schwann cells. **(B)** The expression of classical markers in Schwann cells. **(C)** The cell proportions of Schwann cells between normal skin sample (EN) and earlobe keloid samples (ET). **(D)** GO terms of Biological Process (BP), Cellular Component (CC), and Molecular Function (MF). Here we showed the top 10 categories ordered by *P*-value (<0.05) and gene counts for each ontology. **(E)** The expression of upregulated DEGs in Schwann cells between earlobe keloid samples (ET) and normal skin sample (EN). Here we showed the top 10 DEGs ordered by *P*-value (<0.05) and log2 fold change (avg_log2FC > 0.25). **(F)** GO terms of cellular component (CC) and molecular function (MF) of DEGs in Schwann cells between keloid and normal scars (*P*-value < 0.05).

Schwann cells were defined by canonical neuron markers NRXN1, S100B, SCN7A, and GPM6B (29–31) (Figures 3A,B). We noticed that the cell proportion of SC was more than 10 times larger in earlobe keloid than in normal skin, indicating a potential role of SC in the formation of earlobe keloid (Figure 3C). GO enrichment results showed that the functions of SC were mainly concentrated in two chunks. One was related to the differentiation of neurons and involved in the signal transduction of the nervous system, which was the essential function of SC. At the same time, SC was involved in the ECM structure organization (Figure 3D).

We then explored differentially expressed genes of SC between earlobe keloid and normal skin. We found that the top 10 upregulated DEGs were main collagen genes, like COL11A1 ((Figure 3E), which were associated with the ECM organization and ECM structure constituent, etc. (Figure 3F).

These results suggested that Schwann cells not only participated in signal transduction but also may promote the formation of earlobe keloid by acting on changes in ECM structure.

### Intercellular communication between normal skin and earlobe keloid samples

Next, we explored the cell–cell communication landscape between each cell type and subpopulation, especially the interactions between ECM-related populations and SC in earlobe keloid and normal skin. To better study cell–cell communication mediated by ligand–receptor interactions, we used the R package Cellchat (18).

**FIGURE 4:**
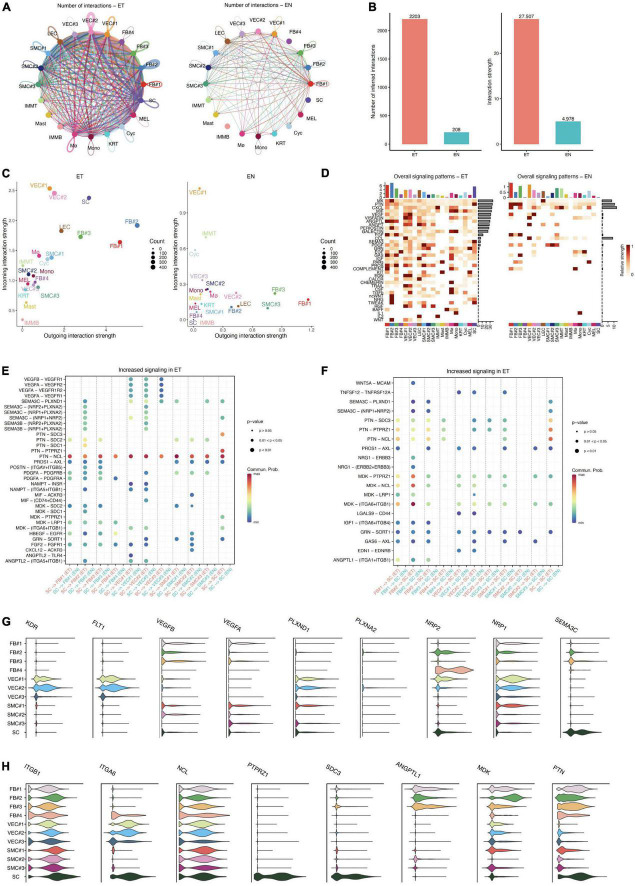
Intercellular communication in normal skin and keloid samples. **(A)** Circos plots show the interactions density between any two cell types in earlobe keloid samples (ET) and normal skin (EN), respectively. **(B)** Bar plots show the number of intercellular communication (left) and interaction strength (right) in earlobe keloid samples (ET) and normal skin (EN), respectively. **(C)** Comparing the outgoing and incoming interaction strength in 2D space by scatter plots to allow identifying the cell populations with significant changes in sending or receiving signals in earlobe keloid samples (ET) and normal skin (EN), respectively. **(D)** The heatmap showed all signaling patterns and the strength of each cell type and subpopulation in earlobe keloid samples (ET) and normal skin (EN), respectively. **(E)** The significant ligand–receptor pairs between ECM-related subpopulations and SC in earlobe keloid samples (ET) and normal skin (EN). Here, SC were as sources and ECM-related subpopulations were as targets. *P*-value < 0.05 was considered significant interactions. **(F)** The significant ligand–receptor pairs between ECM-related subpopulations and SC in earlobe keloid samples (ET) and normal skin (EN). Here, ECM subpopulations were as sources and SC were as targets. *P*-value < 0.05 was considered significant interactions. **(G)** Violin plots showed gene expression level of specific genes in ligand–receptor pairs in ECM-related subpopulations and SC, including SEMA3C and its consistent receptors. **(H)** Violin plots showed gene expression level of specific genes in ligand–receptor pairs in ECM-related subpopulations and SC, including PTN, MDK, and their consistent receptors.

As detailed in Circos plots and bar plots, we found that a denser interaction network in keloid samples compared to that in the normal sample, and the number of intercellular interactions in keloid samples was about 10 times more abundant than that in normal skin (ET: 2203, EN: 208, Figures 4A,B). As previously reported (1, 20, 22, 32), our results also showed that fibroblasts, endothelial cells, and smooth muscle cells are relatively active in the formation of keloids. However, we observed that the interaction of SC with other cells was obviously enhanced in the earlobe keloid, which had not been reported in previous studies (Figure 4A and Supplementary Figure 4), and further suggested that SC may also play an important role during the process of earlobe keloid.

Then, using scatter plots (Figure 4C) to compare the outgoing and incoming interaction strength in 2D space, we observed that fibroblasts and endothelial cells emerge as one of the major sources and targets in earlobe keloid compared to normal skin. FB#1 and FB#2 were the major sources (ligands) in the earlobe keloid, and VEC#1 and VEC#2 were the major targets (receptors) in the earlobe keloid. Compared with signals in normal skin, SC had significant changes in both sending and receiving signals in the earlobe keloid, and mainly interacted with ECM-related subpopulations (Supplementary Figure 4).

To further reveal the outgoing (or incoming) signaling associated with each cell type in keloid and normal skin, respectively, we showed the signaling strength in specific cell populations by heatmap. Overall, the significant signal patterns related to keloid included, but were not limited to MK, MIF, and VEGF (Figure 4D). We observed that the specific and significant outgoing signal patterns of SC in the earlobe keloid were PTN, SEMA3, PDGF, and EGF (Supplementary Figure 5A).

### Identify pathways between extracellular matrix-related subpopulations and Schwann cells contributed to the earlobe keloid

Next, we further identified signaling ligand–receptor pairs related to the process of earlobe keloid, we compared the communication probabilities mediated by ligand–receptor pairs between ECM-related subpopulations and SC (Figure 4E). We found that SC interacted strongly with fibroblast cells and vascular endothelial cells, especially with FB#2 and VEC#2. We observed that the SEMA3 pathway was significant and specific when SCs were the source cells and ECM-related subpopulations were the target cells in a keloid environment (Figure 4E). The signal was further amplified when we identified dysfunctional signaling by comparing DEGs analysis between keloid and normal samples (Supplementary Table 4) and SEMA3C was the main ligand.

SEMA3C protein is a member of the Class 3 semaphorin proteins family, which can interact with neuropilin isoforms (NRP1 and NRP2) and plexin receptors (PLXNA2 and PLXND1) (33, 34), and plays an important role in several respects, such as the neurogenesis (35), cardiac development (36), and promote or inhibit tumor progression (37). SEMA3C can also interact with the VEGF signaling pathways in endothelial cells to regulate cell proliferation and migration and so on (38). SEMAC3 was found to be highly and specifically expressed in SC, according to our findings. Its consistent receptors, such as NRP1, NRP2, PLXND1, and VEGFR, were also highly and specially expressed in ECM-related populations, particularly fibroblasts and vascular endothelial cells (Figure 4G and Supplementary Figure 5B). Thus, we inferred that Schwann cells could interact with fibroblasts and vascular endothelial cells primarily via SEMA3C signaling pathways (SEMA3C_NRP1_PLXNA2, SEMA3C_NRP1_NRP2, SEMA3C_NRP2_PLXNA2, and SEMA3C_PLXND1), which could affect the cells proliferation of fibroblasts and vascular endothelial cells and lead to an increase in their proportion. Besides, Schwann cells could also release VEGF signaling to act on VEC#2, which can also help the cell proliferation of vascular endothelial cells.

Conversely, our results showed that ECM-related subpopulations can promote neurite outgrowth and migration through MK/PTN family (Figure 4F). MK and PTN are a two-member family of heparin-binding neurite outgrowth-promoting factors (39, 40), both of them can ligate with many receptors, such as syndecans (SDC3) (41, 42), integrins (ITGA6) (43), and nucleolin (NCL) (44), which help to promote neurite outgrowth and cell migration in a lot of inflammatory diseases and cancer. Our results showed that PTN was expressed in almost all ECM-related subpopulations, and its consistent receptors SDC3 and ANGPTL1 were highly expressed in SC, but MK was expressed mainly in fibroblasts, especially in FB#2 (Figure 4H and Supplementary Figure 5C). This further confirmed the fibroblast heterogeneity and the importance of FB#2 in earlobe keloid. Thus, we thought that ECM-related subpopulations, mainly the mesenchymal fibroblasts (FB#2), can promote Schwann cell proliferation and migration by the MK/PTN family, which contributed to the development of earlobe keloid.

## Discussion

With the development of single-cell transcriptome technology, it has been gradually applied to the research of human skin diseases (20–22). It can help us understand keloid pathogenesis at the single-cell level and develop more precise therapeutic strategies. Current studies on keloid pathogenesis mainly focus on the mechanisms of fibroblast heterogeneity and hyperproliferation in the skin fibrotic process, as well as related inflammatory responses (20–22). However, the role of SC in keloid pathogenesis has been ignored, especially the relationship between SC and ECM structure.

Therefore, in our study, we performed the scRNA-seq data analysis on earlobe keloid samples and adjacent normal skin samples from the human ear region. We clustered 31,379 cells by UMAP-clustering and partitioned them into 13 major cell types (Figures 1C,D). We defined fibroblasts, vascular endothelial cells, and smooth muscle cells as the ECM populations, as we found these cells were closely related to the structure of ECM (Figure 1G). We further divided ECM-related populations into different subpopulations by distinct signatures, such as specific markers, gene functions, and gene module scores (Figures 2B,C). Referred to previous research (20, 21), we divided fibroblasts into four subpopulations: FB#1: pro-inflammatory fibroblasts, FB#2: mesenchymal fibroblasts, FB#3: secretory-reticular fibroblasts, and FB#4: secretory-papillary fibroblasts (Figure 2A).

We also found that part of vascular endothelial cells, especially VEC#1, could highly express major histocompatibility complex (MHC) genes, such as HLA-DRA and HLA-DRB (Figure 2D). Previous research had reported that vascular endothelial cells can function as immune/inflammation effectors and immune cell mobilizers under a condition (45–47). Therefore, we inferred that during the formation of the earlobe keloid, the expansion of vascular endothelial cells was not only related to the ECM structure but also played an immune role under certain stimulation. However, the specific transformation mechanism of vascular endothelial cells remains to be further studied.

**FIGURE 5:**
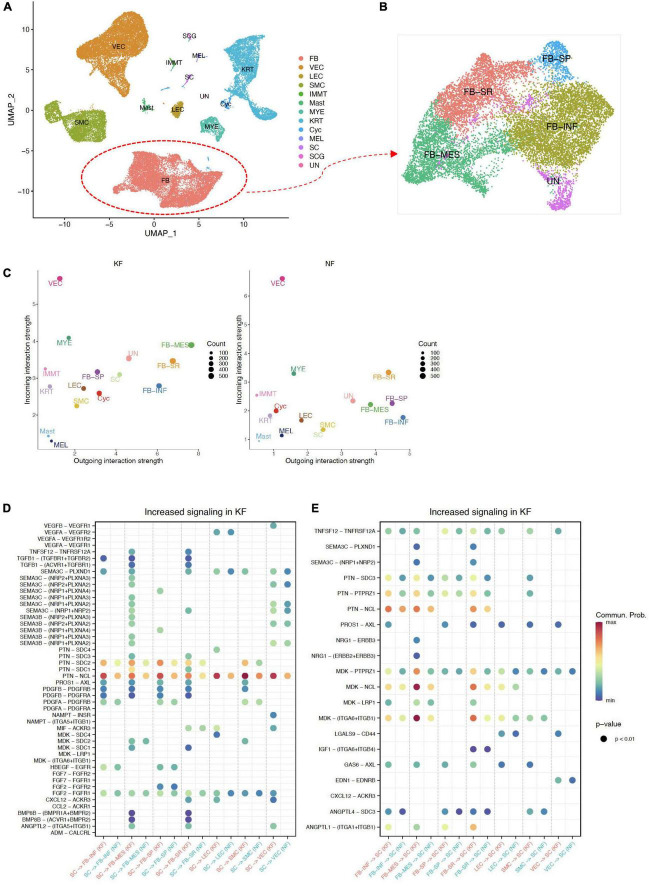
Interactions between ECM-related populations and SC in chest/back keloids. **(A)** UMAP plots of all cells in chest/back keloids, the clustering resolution was 1.5. **(B)** UMAP plots of fibroblasts in chest/back keloids, the clustering resolution was 1.0. **(C)** Comparing the outgoing and incoming interaction strength in 2D space by scatter plots to allow identify the cell populations with significant changes in sending or receiving signals in chest/back keloids (KF) and normal skin (NK), respectively. **(D)** The significant ligand–receptor pairs between ECM-related subpopulations and SC in chest/back keloids (KF) vs. normal skin (NK). Here, SC were as sources. *P*-value < 0.05 was considered significant interactions. **(E)** The significant ligand–receptor pairs between ECM-related subpopulations and SC in chest/back keloids (KF) vs. normal skin (NK). Here, SC were as targets. *P*-value < 0.05 was considered significant interactions.

Our results also verified that Schwann cells were increased significantly in the earlobe keloid (Figure 2A), and actively interacted with ECM-related subpopulations by both sending and receiving signals in the earlobe keloid (Figure 4C and Supplementary Figure 4). Signaling ligand–receptor pairs revealed that SC could influence cell proliferation of ECM-related subpopulations via SEMA3C signaling pathways and VEGF signaling pathways. Conversely, ECM-related subpopulations could also promote SC proliferation and migration by MK/PTN family, especially by the mesenchymal fibroblasts (Figures 5A–D). We noticed that Matrin et al. (48) also found that keloidal Schwann cells contribute to the formation of the extracellular matrix, which could also further support our results. However, they did not further propose the relevant pathways of Schwann cells affecting ECM. Our results could supplement the role of Schwann cells in keloid formation and contribute to comprehensively studying the pathogenesis of keloids.

In addition, in order to explore whether the pathogenesis of different kinds of keloids is site-specific, we compared the difference in interactions between ECM-related subpopulations and SC in different kinds of keloids, including earlobe keloid, back keloid, and chest keloid. The scRNA-seq data of other keloids were downloaded from the GEO database under accession codes “GSE163973” (20). The downloaded data were processed using the above methods. All cells were clustered and partitioned into 13 major cell types by classic markers (Figure 5A and Supplementary Figure 6A). Fibroblasts could also be divided into four main subpopulations: FB-INF: pro-inflammatory fibroblasts, FB-MES: mesenchymal fibroblasts, FB-SR: secretory-reticular fibroblasts, and FB-SP: secretory-papillary fibroblasts (Figure 5B and Supplementary Figure 6B). We found that mesenchymal fibroblasts played an important role in any keloids (Figure 5D). And interactions between SC and ECM-related subpopulations were mainly through SEMA3C signaling pathways and MK/PTN family in both earlobe keloids and chest/back keloids. However, the VEGF signaling pathways were not the main ligand of SC in chest/back keloids. Besides, we found an increase in TGFβ–TGFβ receptor interactions in chest/back keloids compared to earlobe keloid (Figures 5E,F). Our results indicated that the interactions between SC and ECM-related subpopulations had different signaling pathways in different kinds of keloids, which also indicated that the keloid pathogenesis was site-specific.

However, there were still some limitations in our study. First, we lacked sufficient control samples. Therefore, in order to test the accuracy of our data, we verified our data by comparing them with other published single-cell transcriptome data. Second, we will need to further verify the reliability of our results through experiments in future. For example, we can regulate the SEMA3C pathway to observe the development of earlobe keloid formation and help us to better understand the pathogenesis of keloids.

In a word, based on the single-cell transcriptomic map of earlobe keloid, we characterized ECM-related populations and Schwann cells at the single-cell level. We first revealed the role of interactions between ECM-related populations and Schwann cells in the process of earlobe keloid by some main signal pathways. We also revealed that the pathogenesis of different kinds of keloids was site-specific. These findings may help us better understand the pathogenesis of keloids and provide more targeted treatment strategies for different kinds of keloids.

## Data availability statement

The data presented in this study are deposited in the Genome Sequence Archive (GSA) for Human database, accession number: HRA002631. All other relevant data can also be visualized within the article and its Supplementary material or from the corresponding author upon reasonable request.

## Ethics statement

The studies involving human participants were reviewed and approved by the Medical and Ethics Committees of Shenzhen People’s Hospital. The patients/participants provided their written informed consent to participate in this study.

## Author contributions

ZW, QZ, and CZ contributed to the conception and design of the study. YW and SD organized the database and performed the statistical analysis. TG, XM, and DD did the experiment and constructed the single-cell library. YW wrote the first draft of the manuscript. TG and SD revised the manuscript. ZW, QZ, and CZ contributed to the edit and review the manuscript. All authors approved the submitted version.

## Supplementary material

Supplementary Figure 1 Quality control for each sample is based on violin plots. nFature_RNA: the number of genes whose expression is greater than 0 detected in one cell, nCount_RNA: the total gene expression in one cell, percent. mt: percentage of mitochondrial gene expression in one cell, percent. HB: percentage of red blood gene expression in one cell.

Supplementary Figure 2 Two thousand HVGs of merged samples. Top 10 HVGs were labeled on the scatter plot (right).

Supplementary Figure 3 **(A)** UMAP plots of all cells in normal skin sample (ENC07) and earlobe keloid samples (ETB12, ETC02, ETC08, ETD05, and ETF06). **(B)** Dot plot showed the classical markers of all cell types, including subpopulations. Dot size presents the proportion of cells within the group expressing each gene, and dot color is related to its expression level.

Supplementary Figure 4 Network interactions of each cell type in earlobe keloid. Interactions between SC and other cell types were amplified.

Supplementary Figure 5 **(A)** The heatmap showed outgoing and incoming signaling patterns and the strength of each cell type and subpopulation in earlobe keloid samples (ET) and normal skin (EN). **(B)** Violin plots showed gene expression level of specific genes in ligand–receptor pairs in all cell types, including SEMA3C and its consistent receptors. **(C)** Violin plots showed gene expression level of specific genes in ligand–receptor pairs in all cell types, including PTN, MDK, and their consistent receptors.

Supplementary Figure 6 **(A)** Heatmap showed the expression of specific genes in each cell type in chest/back keloids. **(B)** Dot plot showed the classical markers of each fibroblast subpopulations.
