# Supplementary figures and images for: Single-cell RNA-seq reveals the communications between extracellular matrix-related components and Schwann cells contributing to the earlobe keloid formation
Source: Front Med (Lausanne). 2022 Oct 26;9:1000324. doi: 10.3389/fmed.2022.1000324 (PMC9643690; doi:10.3389/fmed.2022.1000324)

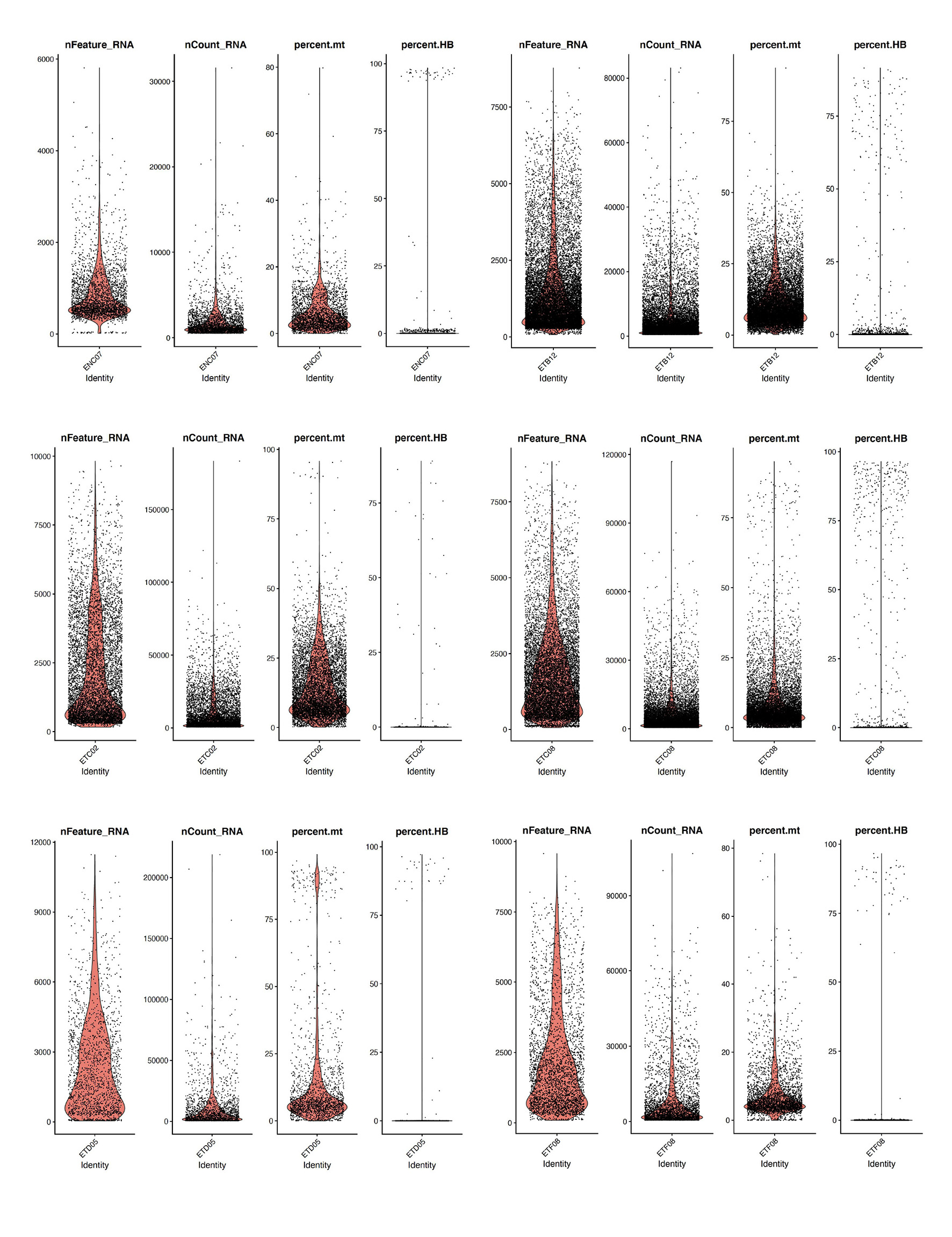

Supplement: Supplementary Figure 1 — Quality control for each sample is based on violin plots. nFature_RNA: the number of genes whose expression is greater than 0 detected in one cell, nCount_RNA: the total gene expression in one cell, percent. mt: percentage of mitochondrial gene expression in one cell, percent. HB: percentage of red blood gene expression in one cell. [file Image_1.JPEG]

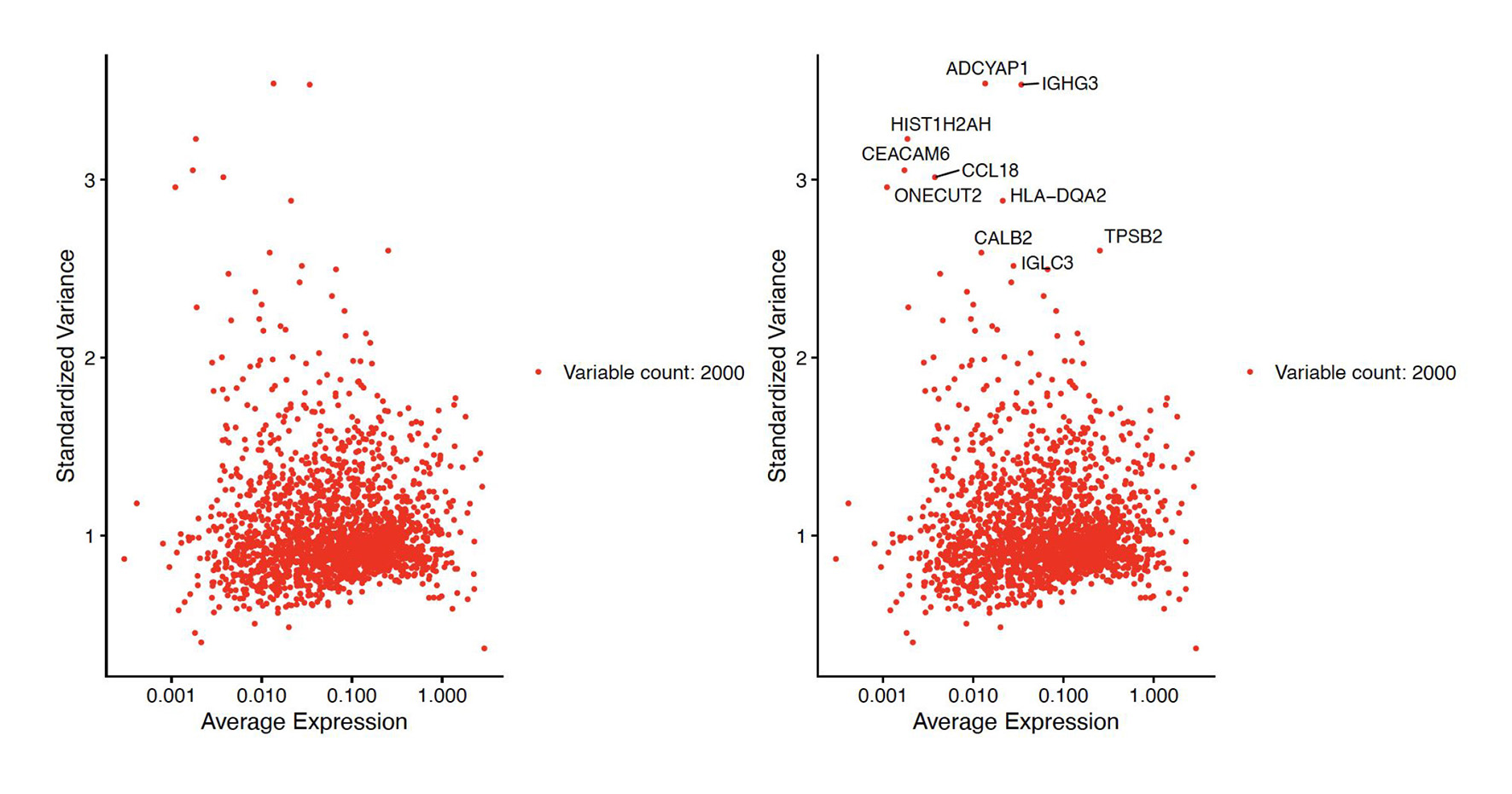

Supplement: Supplementary Figure 2 — Two thousand HVGs of merged samples. Top 10 HVGs were labeled on the scatter plot (right). [file Image_2.JPEG]

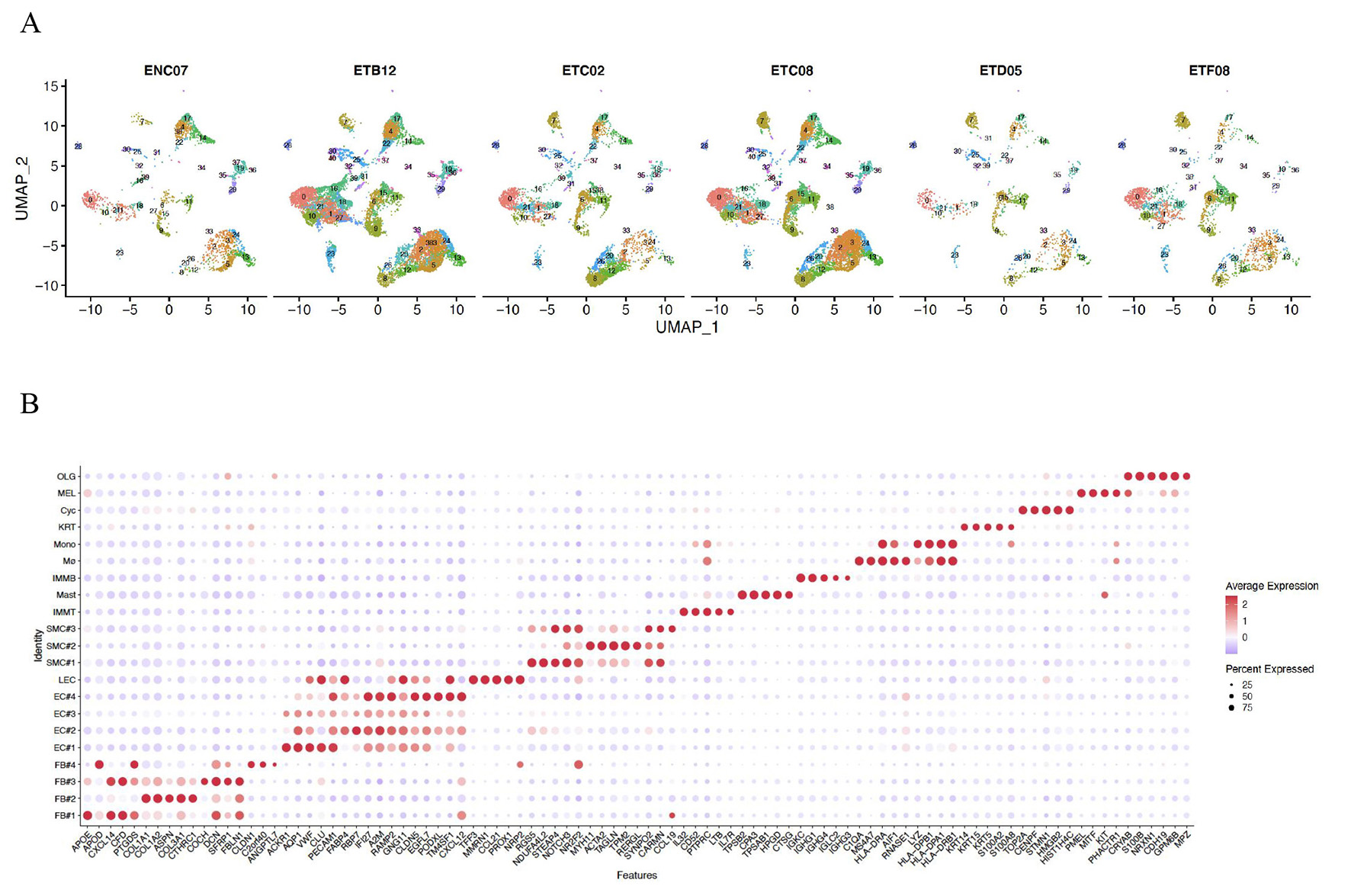

Supplement: Supplementary Figure 3 — (A) UMAP plots of all cells in normal skin sample (ENC07) and earlobe keloid samples (ETB12, ETC02, ETC08, ETD05, and ETF06). (B) Dot plot showed the classical markers of all cell types, including subpopulations. Dot size presents the proportion of cells within the group expressing each gene, and dot color is related to its expression level. [file Image_3.JPEG]

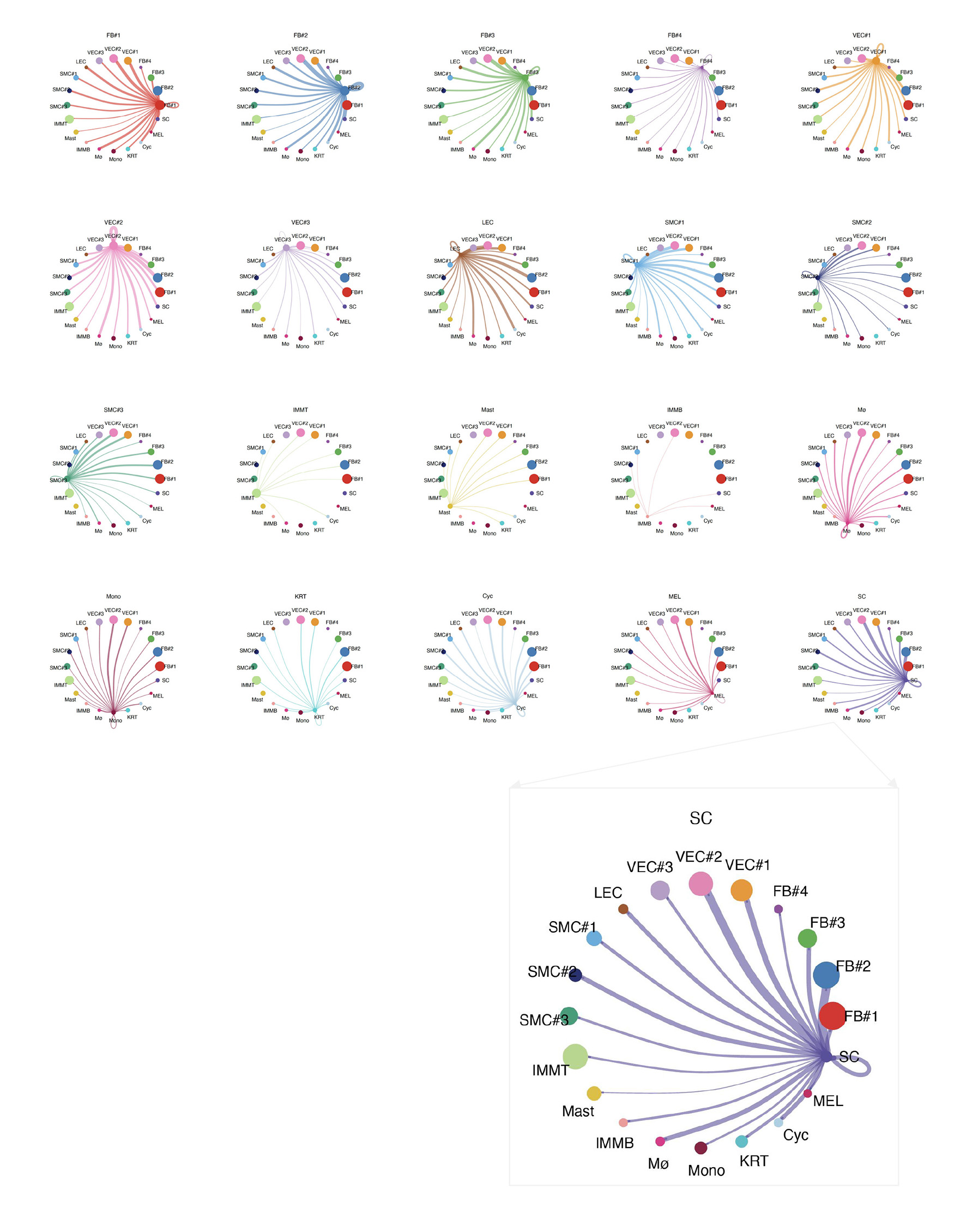

Supplement: Supplementary Figure 4 — Network interactions of each cell type in earlobe keloid. Interactions between SC and other cell types were amplified. [file Image_4.JPEG]

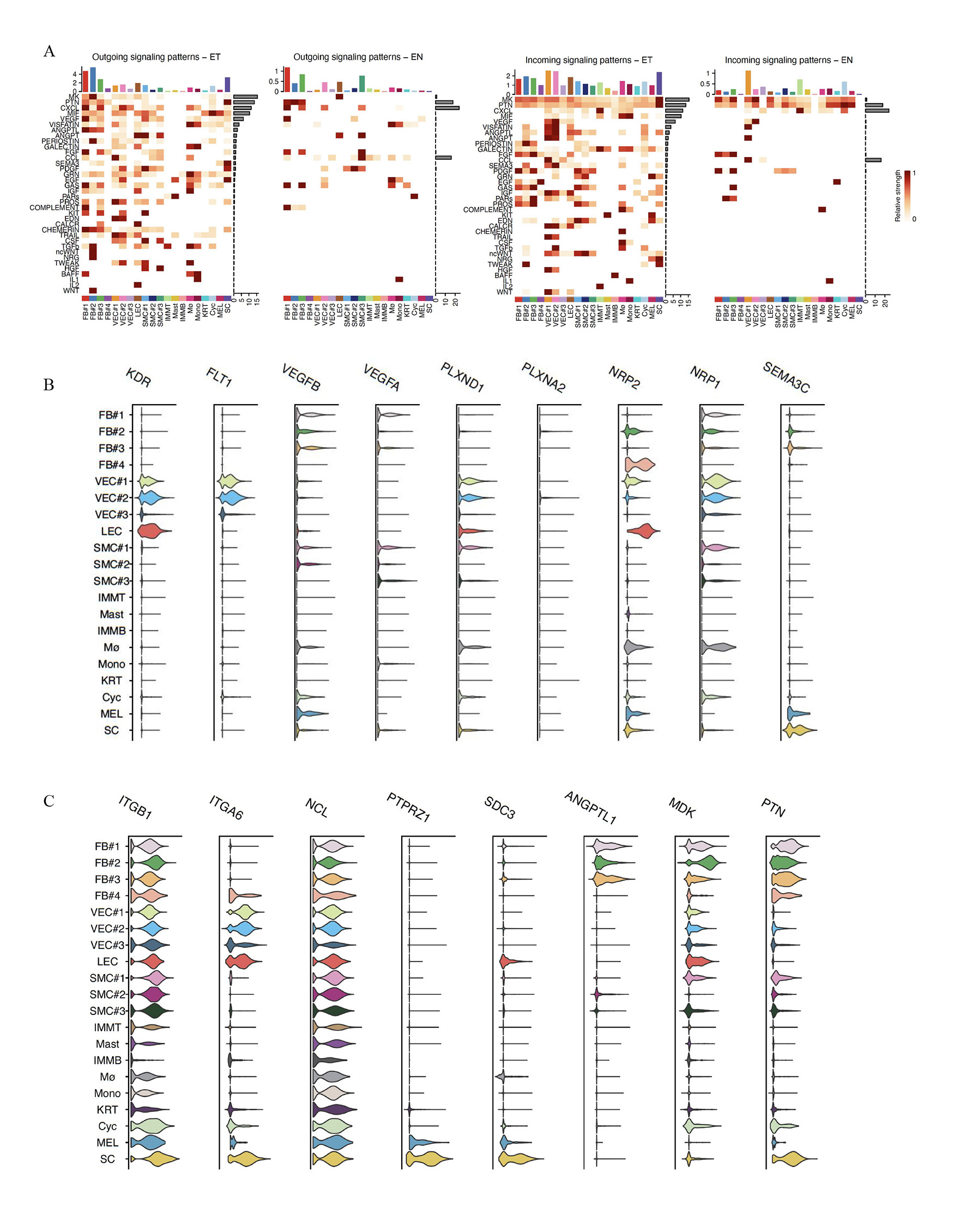

Supplement: Supplementary Figure 5 — (A) The heatmap showed outgoing and incoming signaling patterns and the strength of each cell type and subpopulation in earlobe keloid samples (ET) and normal skin (EN). (B) Violin plots showed gene expression level of specific genes in ligand–receptor pairs in all cell types, including SEMA3C and its consistent receptors. (C) Violin plots showed gene expression level of specific genes in ligand–receptor pairs in all cell types, including PTN, MDK, and their consistent receptors. [file Image_5.JPEG]

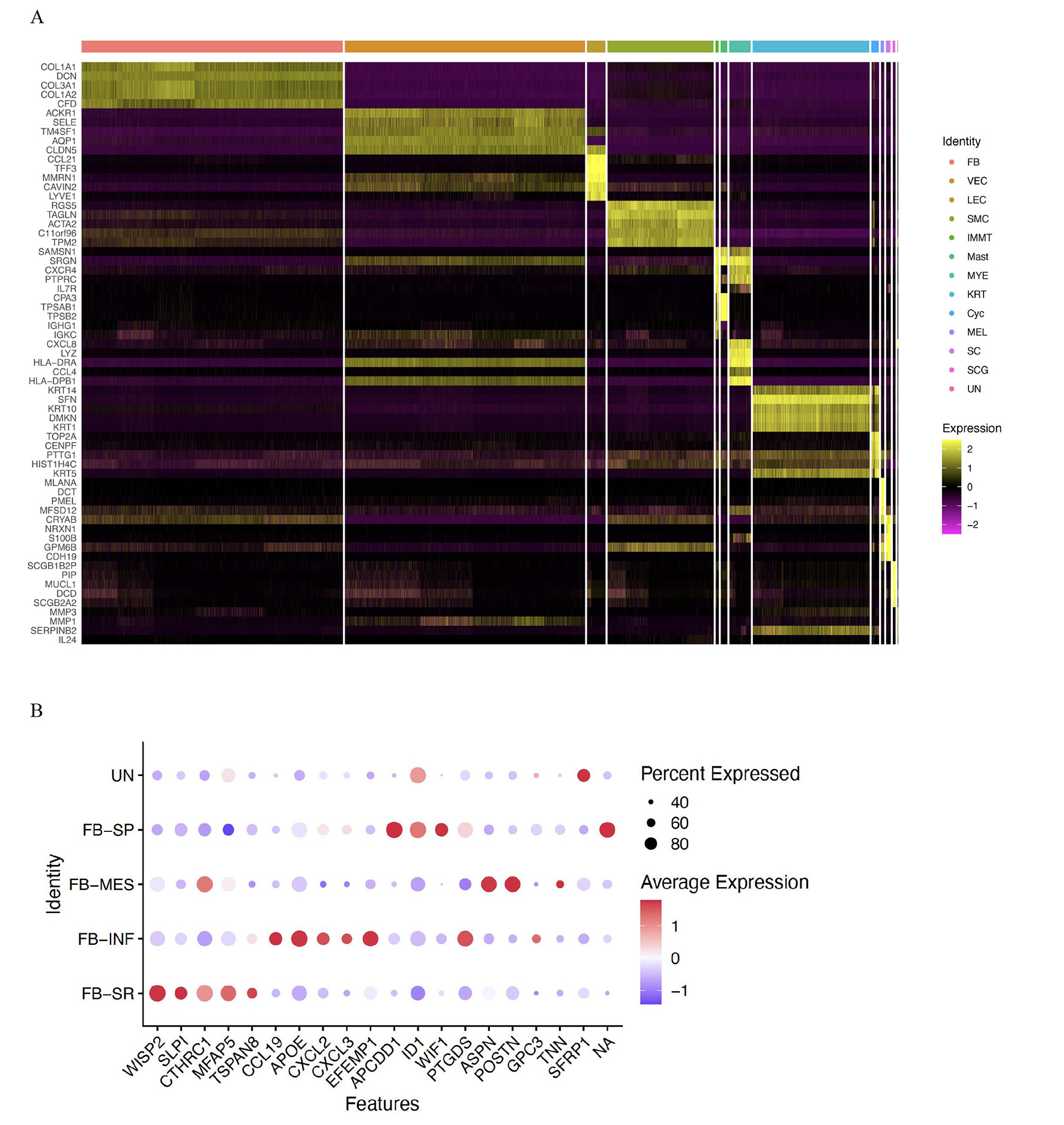

Supplement: Supplementary Figure 6 — (A) Heatmap showed the expression of specific genes in each cell type in chest/back keloids. (B) Dot plot showed the classical markers of each fibroblast subpopulations. [file Image_6.JPEG]
